# Bufotenine is able to block rabies virus infection in BHK-21 cells

**DOI:** 10.1186/1678-9199-20-45

**Published:** 2014-10-13

**Authors:** Hugo Vigerelli, Juliana Mozer Sciani, Carlos Jared, Marta Maria Antoniazzi, Graciane Maria Medeiros Caporale, Andréa de Cássia Rodrigues da Silva, Daniel C Pimenta

**Affiliations:** Laboratory of Biochemistry and Biophysics, Butantan Institute, São Paulo, SP Brazil; Laboratory of Serology, Pasteur Institute, São Paulo, SP Brazil; Laboratory of Cell Biology, Butantan Institute, São Paulo, SP Brazil

**Keywords:** Rabies, *Rhinella*, Bufotenine, Alkaloids, Toxins, Mass spectrometry

## Abstract

**Background:**

Rabies is a fatal zoonotic neglected disease that occurs in more than 150 countries, and kills more than 55.000 people every year. It is caused by an enveloped single stranded RNA virus that affects the central nervous system, through an infection initiated by the muscular nicotinic acetylcholine receptor, according to many authors. Alkaloids, such as acetylcholine, are widespread molecules in nature. They are present in numerous biological fluids, including the skin secretion of many amphibians, in which they act (together with proteins, peptides and steroids) as protection agents against predators and/or microorganisms. Among those amphibians that are rich in alkaloids, there is the genus *Rhinella*.

**Methods:**

Bufotenine was isolated from *Rhinela jimi* skin secretion after a liquid-liquid partition (H_2_O:CH_2_Cl_2_) and reversed phase high-performance liquid chromatography analyses (RP-HPLC). Bufotenine was also extracted from seeds of *Anadenanthera colubrina* in acetone solution and purified by RP-HPLC, as well. Structural characterization was performed by mass spectrometry and nuclear magnetic resonance analyses. Cytotoxic tests of bufotenine were performed over baby hamster kidney (BHK-21) cells using MTT test. For the antiviral activity, *Rabies virus* strain Pasteur vaccine (PV) was used on fluorescence inhibition test and fluorescent foci inhibition test, with both simultaneous and time course treatment of the cells with the virus and bufotenine.

**Results:**

In the present work we describe the effects of bufotenine, obtained either from toads or plants, that can inhibit the penetration of rabies virus in mammalian cells through an apparent competitive mechanism by the nicotinic acetylcholine receptor. Moreover, this inhibition was dose- and time-dependent, pointing out to a specific mechanism of action.

**Conclusions:**

This work do not present or propose bufotenine as a drug for the treatment of rabies due to the hallucinogen and psychotropic effects of the molecule. However, continued studies in the elucidation of the antiviral mechanism of this molecule, may lead to the choice or development of a tryptamine analogue presenting potential clinical use.

**Electronic supplementary material:**

The online version of this article (doi:10.1186/1678-9199-20-45) contains supplementary material, which is available to authorized users.

## Background

According to the World Health Organization (WHO), rabies is a zoonotic neglected disease that occurs in more than 150 countries and territories, killing more than 55,000 people every year, mostly in Asia and Africa [1]. It is caused by a virus that affects the central nervous system and, once symptoms have developed, is nearly always fatal [2, 3]. Different *in vitro* experiments showed evidences that some cells receptors, such as the muscular form of the nicotinic acetylcholine receptor (nAChR), are capable of participating on the entry of rabies virus into cells [4–7]. Other molecules – including the p75 neutrophin, the neuronal cell adhesion molecule (NCAM) and glicosides of the cell membrane – are also capable of participating in the entry of rabies virus into cells, maybe involved in different stages of the infection [8]. Conformational studies on an internal tetrapeptide of rabies virus glycoprotein (Asn^194^-Ser^195^-Arg^196^-Gly^197^) considered this to be an essential part of the binding site of the virus to the acetylcholine receptor and have demonstrated that the side chains of asparagine and arginine can, apparently, mimic the acetylcholine spatial structure, being responsible for binding the virus to the acetylcholine receptor [9].

The skin secretion of some amphibians, on the other hand, is rich in alkaloids [10]. Not only that, but also proteins, peptides, steroids and amines that act as chemical protection agents against predators and/or microorganisms, such as fungi and bacteria [8, 11]. Among those amphibians that are rich in alkaloids, there is the genus *Bufo* that was recently split into *Bufo* in the Old World and *Rhinella* in the New World [12]. Their secretion contain a large number of alkaloids including bufotenine – a tryptamine alkaloid used as a defense mechanism due to its toxicity – that is also found in the Leguminosae family [13–16].

Taking into account that the rabies virus can bind to the nicotinic acetylcholine receptor, and that other alkaloids (nicotine, lobeline, cytisine, anabasine etc.) can also bind to these receptors, the aim of this study was to evaluate the effects of bufotenine as a possible interfering agent in the process of infection of the rabies virus in mammalian cells, using the simplified fluorescent inhibition microtest (SFIMT) and rapid fluorescent focus inhibition test (RFFIT) techniques (with adaptations). Both are serologic tests commonly used in rabies diagnosis that are considered viable tools for the evaluation of new natural compounds as potential antiviral agents.

## Methods

### Reagents

All reagents were of analytical grade and were purchased from Sigma Aldrich (USA), unless otherwise stated.

### Isolation of bufotenine from *Rhinella jimi*skin secretion

The collection and housing of *Rhinella jimi* specimens were performed under license number 15964-1 from the Brazilian Institute of Environment and Renewable Natural Resources (IBAMA). Skins secretions were collected through mechanical stimulation of the parotoid macroglands. A liquid-liquid partition (H_2_O:CH_2_Cl_2_) was performed and the aqueous partition was centrifuged at 4,794 *g* and the supernatant was analyzed by reversed phase high-performance liquid chromatography (RP-HPLC), using a binary HPLC system (20A Prominence, Shimadzu Co, Japan), coupled to a C18 column (ACE®, 250 mm × 7.75 mm). The mobile phase consisted of solvent A [H_2_O: trifluoroacetic acid (TFA); 1000:1] and solvent B [acetonitrile (ACN):H_2_O:TFA; 900:100:1] with a linear gradient of B over A (10% to 70%) in 35 minutes, at a constant flow rate of 1.7 mL.min^-1^ and monitored by UV absorbance at 214 nm. Fractions were manually collected and submitted to mass spectrometry (MS and MS^2^ ESI-IT-TOF) analyses. The fraction containing bufotenine was then purified using a C18 column (ACE®, 250 mm × 4.6 mm), with a linear gradient of B over A 13% to 15% in 15 minutes, at a constant flow rate of 1.1 mL.min^-1^, at 4°C, monitored by UV absorbance at 214 nm. Bufotenine peak was manually collected, dried and submitted to ^1^H-NMR spectroscopic analyses and/or biologic assays.

### Isolation of bufotenine from *Anadenanthera colubrine*seeds

Since bufotenine is a long and well known alkaloid that can be also obtained from other biological sources, such as plants, we chose to purify this molecule from *Anadenanthera colubrina* seeds, which contain up to 2% of the seed dry weight in bufotenine. The seeds were obtained from the legitimate supplier Arbocenter Comércio de Sementes Ltda, Birigui, São Paulo (batch 0019), Brazil. The extraction of bufotenine was performed as described by Stromberg [16], with modifications. Twenty grams of seeds were powdered and subsequently deionized H_2_O and 5 g of sodium carbonate (Na_2_CO_3_) were added until a uniform mixture was obtained. This solution was then lyophilized and ressuspended in 50 mL of acetone (CH_3_COCH_3_). This extract was filtered and concentrated in a rotavapor system, following RP-HPLC analysis, in a C18 column (ACE®, 250 mm × 4.6 mm), with a linear gradient of B over A 0% to 100% in 20 minutes, at a constant flow rate of 1 mL/min and monitored by UV absorbance at 214 nm. Bufotenine peak was manually collected and dried for mass spectrometry and biologic assays.

### Mass spectrometry

Previously dried samples were dissolved into 50% ACN with 0.5% formic acid for MS and/or MS^2^ analyses in an ESI-IT-TOF mass spectrometer system (Shimadzu Co., Japan). Typically, 3 μL of samples were manually injected in a Rheodyne injector under a constant flow of 50 μL/min. The interface voltage used was 4.5 kV and detector voltage, 1.76 kV, with source temperature of 200°C. Data were acquired under positive mode and instrument control, data acquisition and processing were performed by the LCMS solution software (Shimadzu Co., Japan).

### NMR analyses

NMR screening was used to confirm the bufotenine structure from *Rhinella jimi* skin secretion. ^1^H NMR spectra were recorded on Bruker 500 MHz spectrometer (Bruker Co., Germany), with samples diluted in deuterated chloroform and achievement of 128 scans. The results were processed in TopSpin 1.3 software (Bruker Co., Germany).

### Cells and viruses

Baby hamster kidney (BHK-21) cells (ATCC® CCL – 100) were cultured in Eagle’s minimum essential medium, supplemented with 10% fetal bovine serum (MEM-10), at 37°C under a humidified 5% CO_2_ atmosphere until the formation of the cell monolayer. *Rabies virus* strain Pasteur vaccine (PV), from Pasteur Institute, Brazil, was used to determine the antiviral activity of bufotenine. The titers were determined by plate assay in BHK-21 cells and expressed as 100% infecting dose in cell culture (IDCC100) and 50% focus-forming dose (FFD50) as described by Batista *et al*. [17].

### Cytotoxicity assay

The cytotoxicity evaluation of bufotenine was performed by MTT [3-(4,5-dimethylthiazol-2-yl)-2,5-diphenyltetrazolium bromide] method, according to Takeuchi *et al*. [18] and Mosmann [19], with modifications. Briefly, BHK-21 cells (5 × 10^4^ cells/well) were deposited in 96-well tissue culture microtiter plates with 50 μL of different bufotenine concentrations diluted in MEM-10. For positive control of citotoxicity dimethyl sulfoxid (DMSO) 20% was used and the negative control was MEM-10 alone. After 24 hours at 37°C, under a humidified 5% CO_2_ atmosphere, the medium was removed and 50 μL of MTT (Sigma® 1 mg/mL) solution, prepared in MEM-10, was added to each well and the plates were incubated once more for four hours. After incubation, the MTT solution was removed and 100 μL of dimethyl sulfoxide (DMSO) were added to each well to solubilize the formazan crystals. After gently shaking the plates, the crystals were completely dissolved, and the absorbances were measured by using a spectrophotometer (Molecular Devices®, SpectraMax M2) at 540 nm. The CC_50_ was defined as the cytotoxic concentration of bufotenine that reduced the absorbance of treated cells to 50% when compared to the control.

### Antiviral activity

Rabies virus does not cause cytopathic effects in cells cultured *in vitro*, being necessary some kind of additional test to monitor cell infection, such as tests with antibodies bound to fluorescent substances [20]. For this reason, we choose these two tests adapted from commonly serological tests used for antirabies virus titration, being the fluorescence inhibition a qualitative test, for a better visualization of both the infection and its inhibition, and the fluorescence focus inhibition comprises a quantitative test, used for the statistical analyses.

### Fluorescence inhibition test

This test was based on simplified fluorescent inhibition microtest (SFIMT), acording to Favoretto *et al*. [21], with modifications. Briefly, 50 μL of BHK-21 cells (5 × 10^4^ cells/well) were deposited on 96-well tissue culture microtiter plates with 50 μL of each bufotenine dose (from 0.5 to 4 mg/mL) and 50 μL of PV virus previously diluted at 1:100 (IDCC100). As negative inhibition control, only 50 μL of MEM-10 was added to the cells and as positive inhibition control ketamine was used (Dopalen®, 23.4 μM), according to Lockhart *et al*. [22]. After a 24-hour period of incubation at 37°C under a humidified 5% CO_2_ atmosphere, the medium was removed by suction from all wells and the microplates were cooled on ice. The cells were fixed by adding acetone 80% in water (kept at –20°C). After 15 minutes, the plates were emptied by inversion and dried at 37°C according to Smith *et al*. [23] and Chaves *et al*. [24]. The staining was carried out by adding 40 μL of an optimal dilution of the antirabies fluorescent conjugate [25]. After one hour of incubation, the microtiter plates were washed by immersion in PBS and then in distilled water. The microtiter plates reading was performed in inverted fluorescence microscope (Leica DMIL, 100× magnification) in a qualitative manner based on the fluorescence inhibition compared to negative and positive controls, where there are 100% and 0% of fluorescence, respectively.

### Fluorescent focus inhibition test

This test was based on rapid fluorescent focus inhibition test (RFFIT) acording to Smith *et al*. [23], adapted to microtiter plates as described by Chaves *et al*. [24], with modifications. Briefly, 100 μL of BHK-21 cells (2.5 × 10^4^ cells/well) were deposited on 96-well tissue culture microtiter plates containing different bufotenine doses (3.9, 1.95, 0.97 and 0.48 mg/mL), and 50 μL of PV virus previously diluted at thirty-fold the FFD50 value. As negative inhibition control, only 50 μL of MEM-10 was added to the cells and as positive inhibition control ketamine was used (Dopalen®, 23.4 μM). After a 20-hour period of incubation at 37°C under a humidified 5% CO_2_ atmosphere, the medium was removed by suction from all wells. The procedures of cells fixation and staining were the same described in “Fluorescence inhibition test” section. The microtiter plates reading was quantitatively performed in inverted fluorescence microscope (Leica DMIL, 200× magnification), where each field with fluorescence were counted, being 0 the minimum (positive control) and 18 the maximum (negative control) number of infected fields. The IC_50_ was defined as the inhibition concentration of bufotenine that reduced the number of fields with fluorescent foci to 50% when compared with the negative control.

### Time course study

The time course effect of bufotenine was examined on PV virus with two minor modifications of the fluorescent focus inhibition test. First, to test a possible “protector effect”, bufotenine was added in different times (1, 3 and 6 hours), *prior* to the addition of the virus and, secondly, to test a possible “treatment effect”, bufotenine was added in different times (1, 3 and 6 hours), *after* incubation of cells and PV virus. All the other procedures were the same as described in “Fluorescent focus inhibition test” section.

### Data analysis

The 50% cytotoxic (CC_50_) and inhibition (IC_50_) concentrations were calculated from concentration-effect curves after linear regression analysis. The results represent the mean ± standard error of the mean values of two different experiments (triplicates).

## Results

### RP-HPLC and mass spectrometry analysis

**Figure 1:**
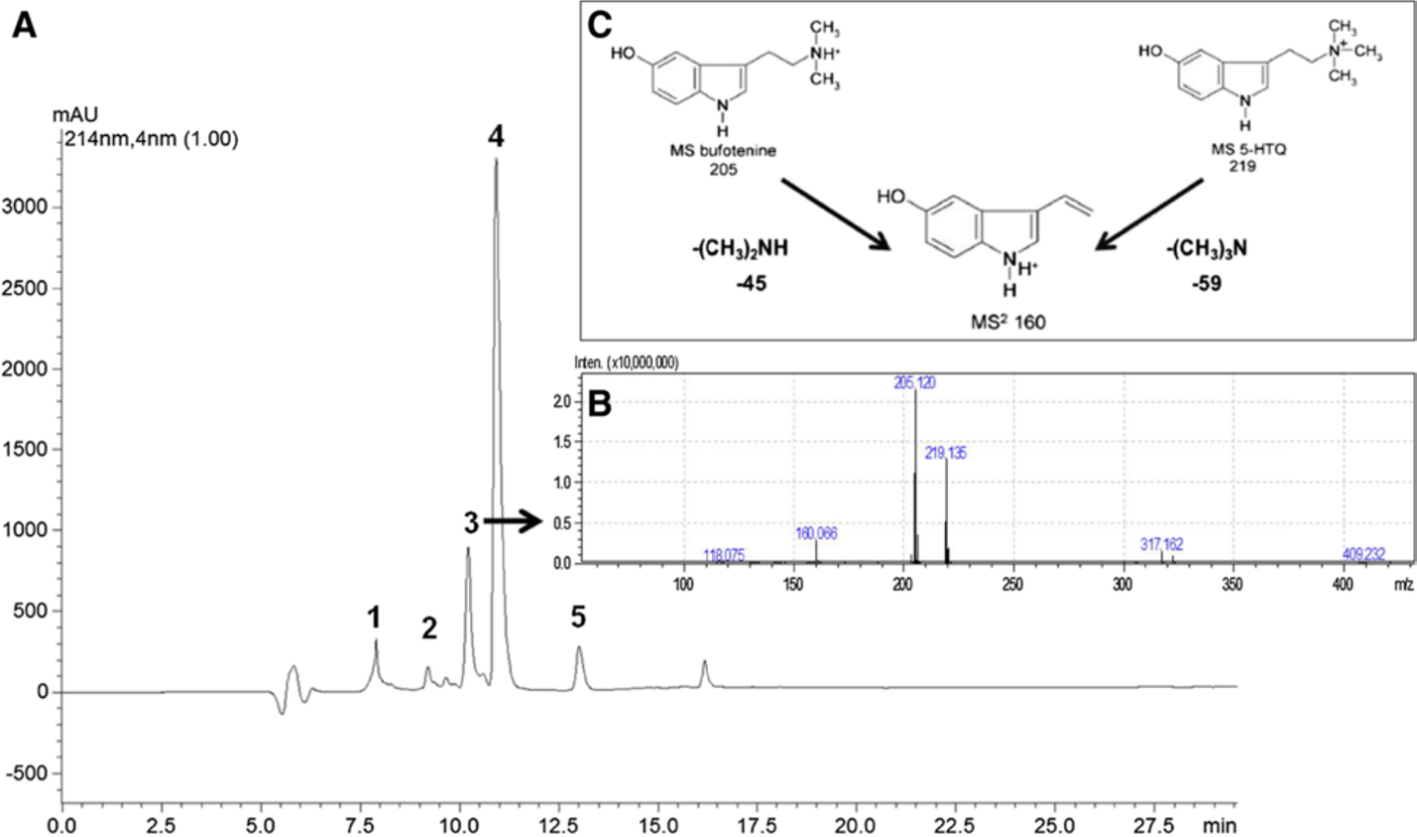
**RP-HPLC analysis of the aqueous partition of** ***Rhinela jimi*** **skin secretion. (A)** RP-HPLC profile of *Rhinella jimi* aqueous partition (*λ* = 214 nm), showing five fractions. **(B)** MS analysis of fraction 3 showing three major molecules of 205, 219 and 160 m/z. **(C)** Fragmentation of indole alkaloids bufotenine (205 m/z) and 5-HTQ (219 m/z), resulting in an ion of 160 m/z.

RP-HPLC analysis of the aqueous partition of *Rhinela jimi* skin secretion showed the presence of five major HPLC peaks (Figure 1A). MS analysis showed that fraction 3 contained the following m/z values: 205, 219 and 160 (Figure 1B). Comparing the MS^2^ analysis with already published data by McClean *et al*. [26] (Figure 1C), it was possible to characterize the two indole alkaloids contained in this fraction: the 205 m/z correspond to N’,N’-dimethyl 5-hydroxytryptamione (bufotenine) and 219 m/z correspond to N’,N’,N’-trimethyl 5-hydroxytryptamine (5-HTQ). The 160 m/z ion is a spontaneous fragmentation of both alkaloids, which also appears on MS^2^ profile (Additional file 1: Figure S1). This two compounds were separated by RP-HPLC yielding pure bufotenine (Additional file 2: Figure S2), as confirmed by NMR analyses (Additional file 3: Figure S3). Bufotenine from *Anadenanthera colubrina* seeds were purified only after one chromatographic step, following the acetone extract, being the major peak on the RP-HPLC chromatogram and appearing pure on mass spectrometric analyses (Additional file 4: Figure S4).

### Cytotoxicity effect on viability of BHK-21 cells

The 3.9 mg/mL dose of *R. jimi* bufotenine used on the virologic tests presented statistically significant cytotoxic effects on the viability of BHK-21 cells (66% of viable cells) when compared to the negative (cells + MEM-10) and positive controls (cells + DMSO 20%) (Additional file 5: Figure S5). Subsequent assays were performed with the seeds bufotenine, due to the high availability. In the MTT test (Additional file 6: Figure S6), it was possible to determine the CC_50_ value (7.6 mg/mL).

### Antiviral activity

- Fluorescence inhibition test

**Figure 2:**
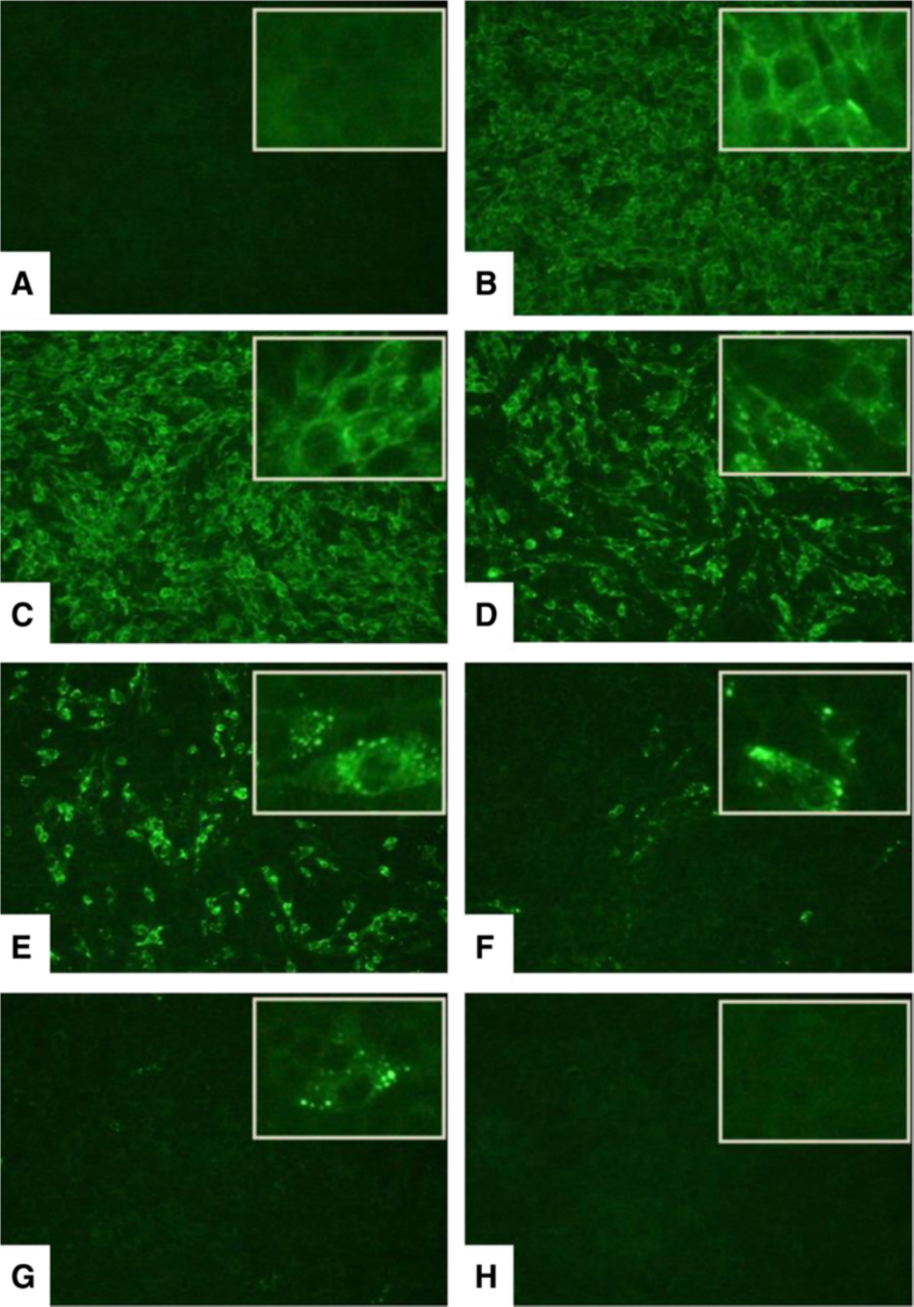
**Effects of bufotenine from** ***Anadenanthera colubrine*** **on fluorescence inhibition test. (A)** positive inhibition control (ketamine); **(B)** negative control (cells + MEM-10); bufotenine concentrations: **(C)** 0.5 mg/mL, **(D)** 1.0 mg/mL, **(E)** 1.5 mg/mL, **(F)** 2.0 mg/mL, **(G)** 2.5 mg/mL, **(H)** 3.0 mg/mL. Magnification 100×. Insert magnification 200 ×.

Bufotenine was able to inhibit the infection significantly, showing dose-response effect when tested at a concentration of 0.5 to 3 mg/mL (Figure 2).

- Fluorescent focus inhibition test

**Figure 3:**
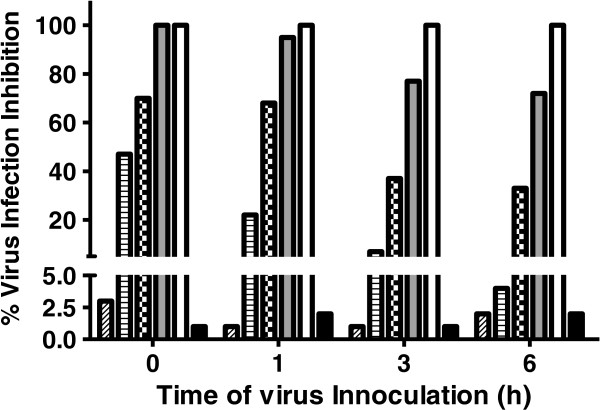
**PV virus inhibition effect of bufotenine on fluorescent focus inhibition test,** **at different times adding PV virus** **(0,** **1,** **3 and 6 hours),** **compared with negative** **(cells +** **MEM-** **10)** **and positive** **(ketamine)** **controls** **(black and white columns,** **respectively)** **.** Bufotenine concentrations: 3.9 mg/mL (gray columns), 1.95 mg/mL (checkered columns), 0.97 mg/mL (striped columns) and 0.48 mg/mL (diagonally striped columns).

**Figure 4:**
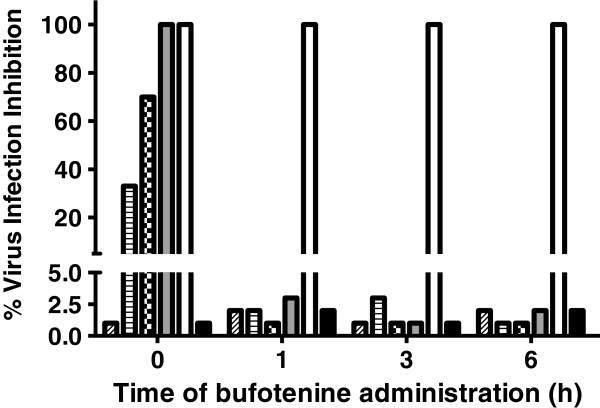
**PV virus inhibition effect of bufotenine added in different times** **(0,** **1,** **3 and 6 hours)** **on fluorescent focus inhibition test,** **compared with negative** **(cells +** **MEM-** **10)** **and positive x** **(ketamine)** **controls** **(black and white columns,** **respectively)** **.** Bufotenine concentrations: 3.9 mg/mL (gray columns), 1.95 mg/mL (checkered columns), 0.97 mg/mL (striped columns) and 0.48 mg/mL (diagonally striped columns).

**Figure 5:**
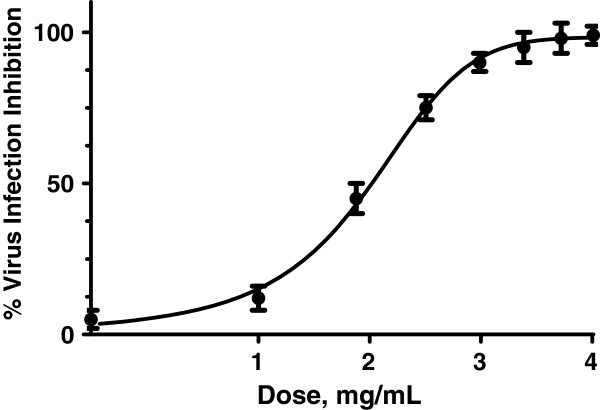
**PV virus inhibition percentage of different bufotenine concentrations on fluorescent focus inhibition test compared with negative** **(cells +** **MEM-** **10)** **and positive** **(ketamine)** **controls.**

Bufotenine was able to inhibit 100% of the infection at 3.9 mg.mL^-1^. It was also possible to observe the dose-response effect. Moreover, there was a time course dependency of the effect regarding the moment of PV virus administration, being bufotenine most effective when added along with the cells and PV virus (time 0, Figure 3). On the other hand, when bufotenine was administered over the time, the molecule was unable to inhibit the infection, regardless of the tested concentration, from one hour onwards (Figure 4). After testing different bufotenine concentrations in the most effective conditions used in the time course study (time 0), it was possible to determine the IC_50_ value of (1.57 ± 0.03) mg.mL^-1^ (Figure 5).

## Discussion

Although bufotenine was isolated “only” in 1920, shamans and mystics have been employing toads’ skin secretions since ancient times as *entheogens*, which means “generating the divine within” [27]. The chemical structure elucidation was published in 1934 by the group of the chemist Heinrich Otto Wieland and one can observe that the molecule is related to psilocin and dimethyltryptamine (DMT), which are known hallucinogens, as well to the neurotransmitter serotonin [28]. Another early report on the molecule is the documentation of its toxicity, by the roman poet Decimus Junius Juvenalis (60-128 AD) in the first century.

In this work, we describe bufotenine as an inhibitor of rabies virus penetration in mammal cells. Initial bufotenine isolation and characterization was performed from the skin secretion of the toad *R. jimi* based on biological effect-driven assays, as previously done by the authors [13, 29–31]. After initial molecular identification and characterization, we have opted for obtaining this molecule from a more abundant source, so the seeds of *Anadenanthera colubrina* were chosen, according to early reports on the isolation of this alkaloid of *Anadenanthera* genus [16].

Bufotenine has been purified, described and synthesized many years ago [32–34]. However, the original isolation process includes several steps of extraction, filtering, partitioning and pH adjustments [16]. In our work, on the other hand, we were able to demonstrate that the use of chromatographic techniques allows for greater efficiency in the purification of this molecule. Moreover, the association with mass spectrometry analyses allow further evaluation of the purity degree, as well as molecular identity confirmation, for the subsequent biological assays. From the toad skin secretion, pure bufotenine could be obtained after two chromatographic steps, and from the seeds, bufotenine was purified after acetone extraction employing only one chromatographic step, which increased the efficiency and the yield in the obtainment of the pure molecule.

There are recent described effects of hydroalcoholic extracts of different parts of *A. colubrina*, such as anti-inflammatory and peripheral antinociceptive activities in rodent models, antimicrobial (anti-*Staphylococcus*) and cutaneous wound healing in rats, among others [35–37]. However, these reports describe biological effects of extracts rather than pure molecules. Although the toxic and hallucinogens effects of bufotenine are widely discussed and studied, no reports about its antiviral activity are known to the best of our knowledge [38–40].

Among the possible described mechanisms of rabies virus penetration known (or postulated), the bufotenine inhibition of rabies virus infection observed in this study appears to be related to the competition to the cells’ receptors, since it could only be observed when simultaneous administration of bufotenine and PV virus was performed (Figure 4). However, the elucidation of this mechanism depends on electrophysiological and/or patch clamp experiments to be performed in the future.

Numerous authors have described that there are high-affinity receptors at host cells specific for rabies virus, which include AChR – and/or associated molecules – that act as receptors. Although it was not possible to unequivocally demonstrate that the inhibitory effect of bufotenine was mediated by its binding to the acetylcholine receptor, the results of this work open important perspectives in the study of the mechanism of action of bufotenine, as well as the elucidation of the mechanisms of the virus pathogenicity. Altogether, our data may aid in the development of 2^nd^ generation active molecules, since rabies is still a disease with no cure prognosis and it kills, every year, thousands of people around the world [1]. Furthermore, bufotenine is cytotoxic, so medicinal chemistry and lead optimization will be required.

## Conclusions

This work do not present or propose bufotenine as a drug for the treatment of rabies for several reasons, including the hallucinogen and psychotropic effects of the molecule. However, continued studies in the elucidation of the antiviral mechanism of this molecule may lead to the choice or development of a tryptamine analogue presenting potential clinical use. These tryptophan derived alkaloids are very interesting building blocks in organic chemistry syntheses [41]. Our next goal would be to evaluate such analogues as possible inhibitors of the viral infection, targeting the dissociation of toxic and psychotropic effects from the antiviral effect.

### Ethics committee approval

The collection and housing of *Rhinella jimi* specimens were performed under license number 15964-1 from the Brazilian Institute of Environment and Renewable Natural Resources (IBAMA).

## Electronic supplementary material

Additional file 1: Figure S1: ESI-IT-TOF MS^2^ fragmentation profile of (A) m/z 205 (bufotenine) and (B) m/z 219 (N’,N’,N’-trimethyl 5-hydroxytryptamine [5HTQ]) molecules. Note the common m/z 160 ion. (DOCX 333 KB)

Additional file 2: Figure S2: Figure showing (A) C18-RP-HPLC profile of the separation of (B) bufotenine and (C) 5HTQ, at 4°C. (DOCX 131 KB)

Additional file 3: Figure S3: Interpretation and annotation of ^1^H-NMR spectrum of bufotenine purified from *R. jimi* skin secretion. (DOCX 113 KB)

Additional file 4: Figure S4: Figure showing (A) ESI-IT-TOF MS profile of bufotenine purified from *A. colubrine* seeds, as represented by (B) the RP-HPLC profile. (DOCX 134 KB)

Additional file 5: Figure S5: Cytotoxicity evaluation of bufotenine, as assayed at the most effective antiviral dose. (DOCX 35 KB)

Additional file 6: Figure S6: Percentage of viable cells after treatment with different bufotenine concentrations compared with negative (cells + MEM-10) and positive (DMSO 20%) controls. (DOCX 46 KB)
