# Supplementary figures and images for: Bufotenine is able to block rabies virus infection in BHK-21 cells
Source: J Venom Anim Toxins Incl Trop Dis. 2014 Oct 13;20:45. doi: 10.1186/1678-9199-20-45 (PMC4203886; doi:10.1186/1678-9199-20-45)

**
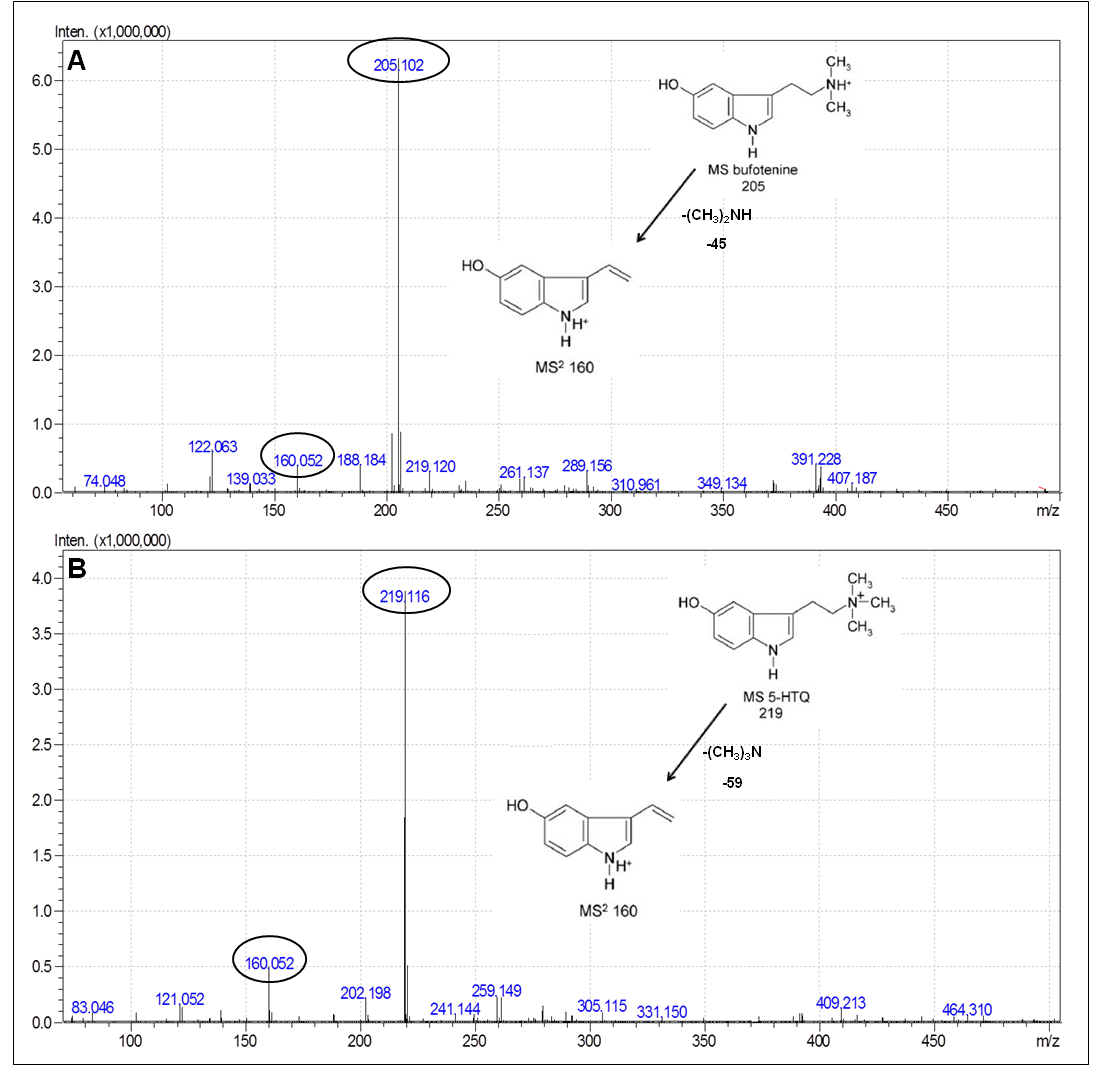
**

Supplement: Supplementary file 1 — Additional file 1: Figure S1: ESI-IT-TOF MS2 fragmentation profile of (A) m/z 205 (bufotenine) and (B) m/z 219 (N’,N’,N’-trimethyl 5-hydroxytryptamine [5HTQ]) molecules. Note the common m/z 160 ion. (DOCX 333 KB) [file 40409_2014_71_MOESM1_ESM.docx]

**
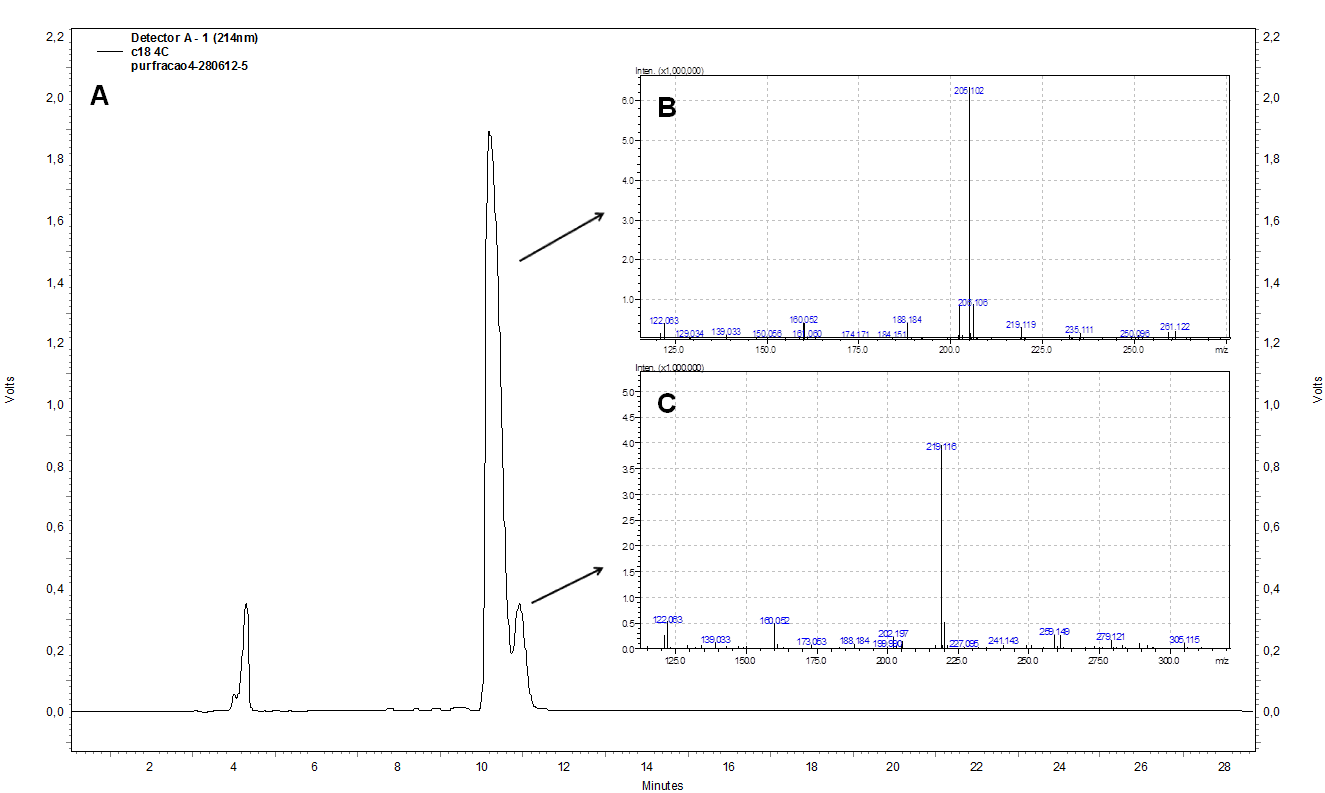
**

Supplement: Supplementary file 2 — Additional file 2: Figure S2: Figure showing (A) C18-RP-HPLC profile of the separation of (B) bufotenine and (C) 5HTQ, at 4°C. (DOCX 131 KB) [file 40409_2014_71_MOESM2_ESM.docx]

**
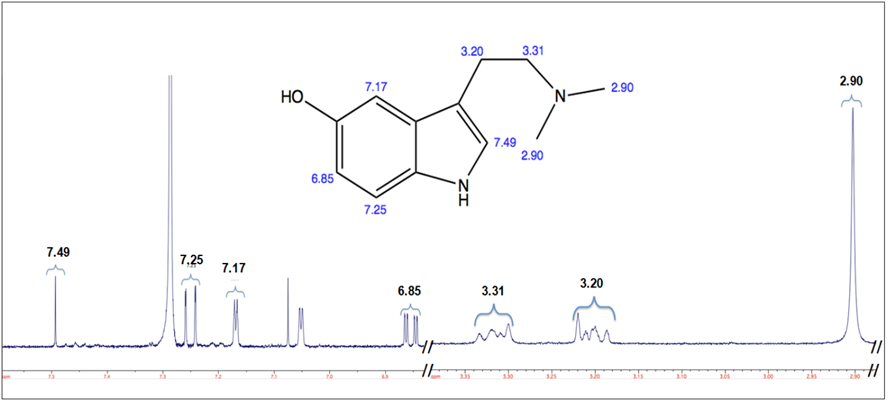
**

Supplement: Supplementary file 3 — Additional file 3: Figure S3: Interpretation and annotation of 1H-NMR spectrum of bufotenine purified from R. jimi skin secretion. (DOCX 113 KB) [file 40409_2014_71_MOESM3_ESM.docx]

**
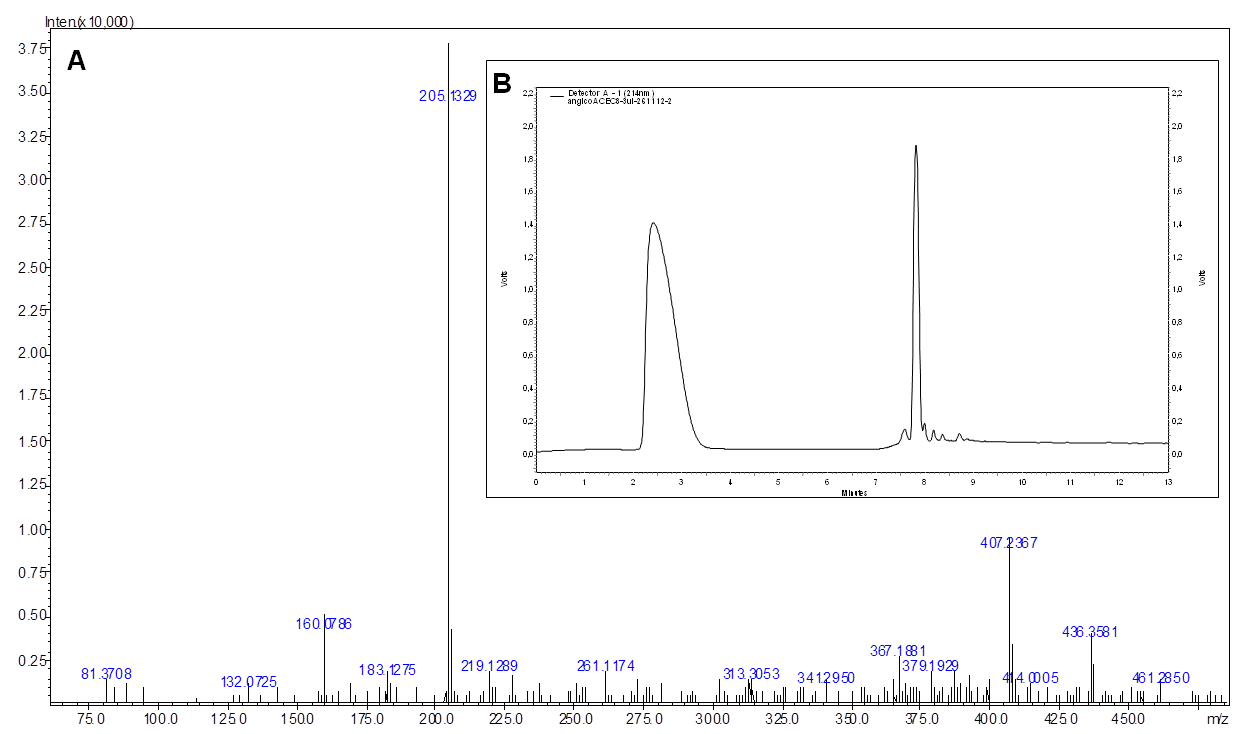
**

Supplement: Supplementary file 4 — Additional file 4: Figure S4: Figure showing (A) ESI-IT-TOF MS profile of bufotenine purified from A. colubrine seeds, as represented by (B) the RP-HPLC profile. (DOCX 134 KB) [file 40409_2014_71_MOESM4_ESM.docx]

**
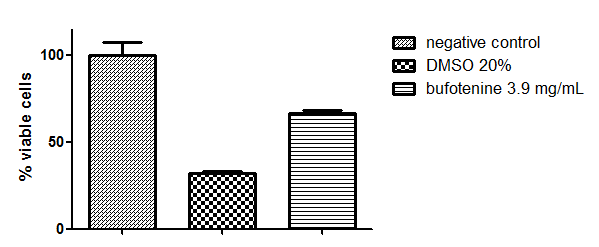
**

Supplement: Supplementary file 5 — Additional file 5: Figure S5: Cytotoxicity evaluation of bufotenine, as assayed at the most effective antiviral dose. (DOCX 35 KB) [file 40409_2014_71_MOESM5_ESM.docx]
