# Supplementary material for: Bufotenine is able to block rabies virus infection in BHK-21 cells
Source: J Venom Anim Toxins Incl Trop Dis. 2014 Oct 13;20:45. doi: 10.1186/1678-9199-20-45 (PMC4203886; doi:10.1186/1678-9199-20-45)
Supplement: Supplementary file 2 [file 40409_2014_71_MOESM6_ESM.docx]

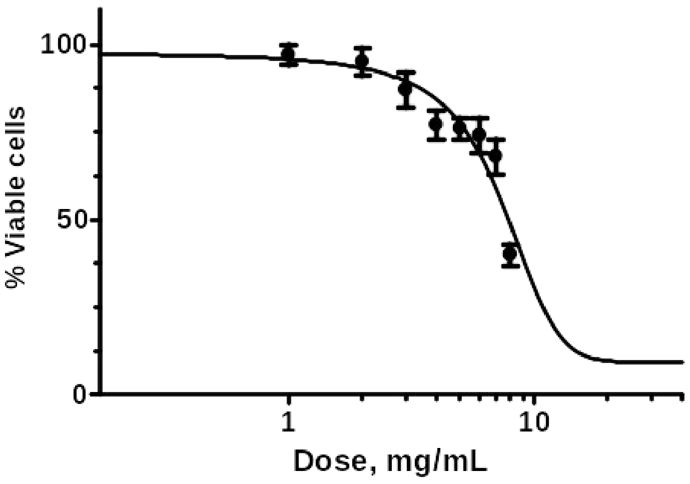

100
50
0
% Viable cells
1
10
Dose, mg/mL
